# Therapeutic potential of photoimmunotherapy in solid tumors expressing CD98 heavy chain

**DOI:** 10.1038/s41598-026-57005-3

**Published:** 2026-06-09

**Authors:** Citra R. A. P. Palangka, Naoya Kondo, Ayaka Kanai, Hirofumi Hanaoka

**Affiliations:** Fundamental Technology Development Division, Near InfraRed Photo-Immunotherapy Institute, Kansai Medical University, Osaka, 573-1010 Japan

**Keywords:** Near-infrared photoimmunotherapy, CD98hc, IGN523-IR700, Solid tumors, Cancer, Immunology, Oncology

## Abstract

Near-infrared photoimmunotherapy (NIR-PIT) is an emerging targeted cancer therapy that uses an antibody–photoabsorber conjugate. In this study, we evaluated the therapeutic potential of NIR-PIT targeting CD98 heavy chain (CD98hc), a transmembrane glycoprotein that is highly expressed in various solid tumors and is associated with poor prognosis. We prepared a conjugate of the clinically tested anti-CD98hc antibody IGN523 and photoabsorber IRDye700DX (IGN523-IR700). The therapeutic efficacy was assessed in vitro using four CD98hc-expressing cancer cell lines and in an in vivo tumor xenograft mouse model. In vitro, IGN523-IR700 binds to cancer cells and induces rapid and light dose-dependent cell death upon near-infrared light exposure. In the in vivo model, intravenous administration of IGN523-IR700 followed by tumor-directed light irradiation resulted in significant tumor growth inhibition compared with that in the control groups. These results demonstrate that CD98hc is a viable and effective target for NIR-PIT. Given its widespread expression, targeting CD98hc may significantly broaden the applicability of NIR-PIT to diverse cancer types.

**Supplementary Information:**

The online version contains supplementary material available at 10.1038/s41598-026-57005-3.

## Introduction

Near-infrared photoimmunotherapy (NIR-PIT) is an emerging targeted cancer treatment that combines monoclonal antibodies with the photoabsorber IRDye700DX (IR700), enabling the selective destruction of tumor cells upon exposure to NIR light. In NIR-PIT, upon antibody binding to tumor cell membrane targets, irradiation with 690-nm light induces IR700 insolubilization via a chemical structural change, followed by protein aggregation, leading to the physical disruption of cell membranes. This method provides high tumor specificity, minimal off-target toxicity, and induces immunogenic cell death, making it a promising strategy for cancer therapy^1–3^. Clinically, NIR-PIT has been extensively developed using antibodies against the epidermal growth factor receptor (EGFR), and EGFR-targeted NIR-PIT has been approved for the treatment of head and neck cancers in Japan. Additionally, preclinical experiments have shown that NIR-PIT can treat various cancers by targeting molecules, such as HER2, PSMA, CEA, GPC3, mesothelin, CD25, CD20, PD-L1, CD44, CD133, laminin, and MUC1^4–13^. Given the mechanism of NIR-PIT and that all these targets are expressed on the cancer cell membrane surface, antibody binding to these targets is essential for therapeutic efficacy. However, considering the heterogeneous nature of cancer, cells lacking target expression are not subject to the direct cytotoxic effects of NIR-PIT; therefore, pretreatment evaluation of target expression on the cancer cell surface is important for predicting therapeutic response. However, determining the expression of the newly proposed target molecules in individual patients remains a major challenge.

To address this challenge^1^, we integrated NIR-PIT with positron emission tomography (PET) imaging and focused on L-type amino acid transporter 1 (LAT1). LAT1 mediates the transport of essential amino acids across cancer cell membranes and is markedly upregulated in many malignancies. Amino acid analog PET tracers, such as L-3-[^18^F]-fluoro-α-methyl tyrosine and *(S)*-2-amino-3-[3-(2-[^18^F]-fluoroethoxy)-4-iodophenyl]-2-methylpropanoic acid, enable LAT1-based tumor imaging, offering a potential diagnostic companion for therapy selection^14–16^. Therefore, antibody-based LAT1-targeting drugs are promising cancer therapies. However, no antibodies that specifically bind to LAT1 on the cell membrane have been reported, as it has a minimal extracellular domain.

Therefore, we shifted our focus to CD98 heavy chain (CD98hc), a transmembrane glycoprotein with a large extracellular domain that forms a disulfide-linked heterodimer with LAT1 and facilitates LAT1 trafficking and stability at the plasma membrane^17,18^. CD98hc is overexpressed in various solid tumors, including head and neck squamous cell carcinoma^19^, glioblastoma^20^, colon adenocarcinoma^21^, pancreatic ductal adenocarcinoma^22^, non-small cell lung cancer^23^, breast cancer^24^, and brain cancer^25^. Its expression is strongly correlated with tumor progression, metastasis, therapy resistance, and poor prognosis. Importantly, CD98hc is required for the trafficking and stable surface expression of LAT1, with previous studies reporting positive correlations between CD98hc and LAT1 expression (*r* = 0.334–0.598, *p* < 0.001; ρ = 0.633–0.729, *p* < 0.0001)^24,26–31^. These findings suggest that LAT1 PET imaging may provide indirect information on CD98hc abundance and help identify tumors that could be suitable for CD98hc-targeted NIR-PIT.

In this study, we investigated the therapeutic potential of CD98hc-targeted NIR-PIT using the clinically tested anti-CD98hc monoclonal antibody, IGN523, conjugated with IR700. We evaluated its therapeutic efficacy both in vitro and in vivo using tumor models with high CD98hc expression to establish CD98hc as a novel therapeutic target for NIR-PIT.

## Results

### Analysis of IGN523-IR700 and IGN523-FITC

An IGN523-IR700 conjugate with an average of 3.62 IR700 molecules per antibody, was successfully synthesized. An IGN523-FITC conjugate, which was used as a reference conjugate to approximate the binding property of the parental IGN523, had an average of 1.2 FITC molecules per antibody. Purity analysis by size-exclusion chromatography (SEC-HPLC) confirmed that contamination from unreacted free IR700 was less than 1% (Fig. S1).

In the flow cytometry analysis of A549 cells, a greater amount of IGN523-IR700 was required than IGN523-FITC to reach a plateau in fluorescence signal (Fig. S2). Based on the flow cytometry results, the Kd values for IGN523-IR700 and IGN523-FITC were 198 and 1.71 nM, respectively. This suggests that binding affinity would decrease due to IR700 conjugation; however, IGN523-IR700 still showed high binding when the amount of antibody was increased.

### Cell binding of IGN523-IR700

**Fig. 1:**
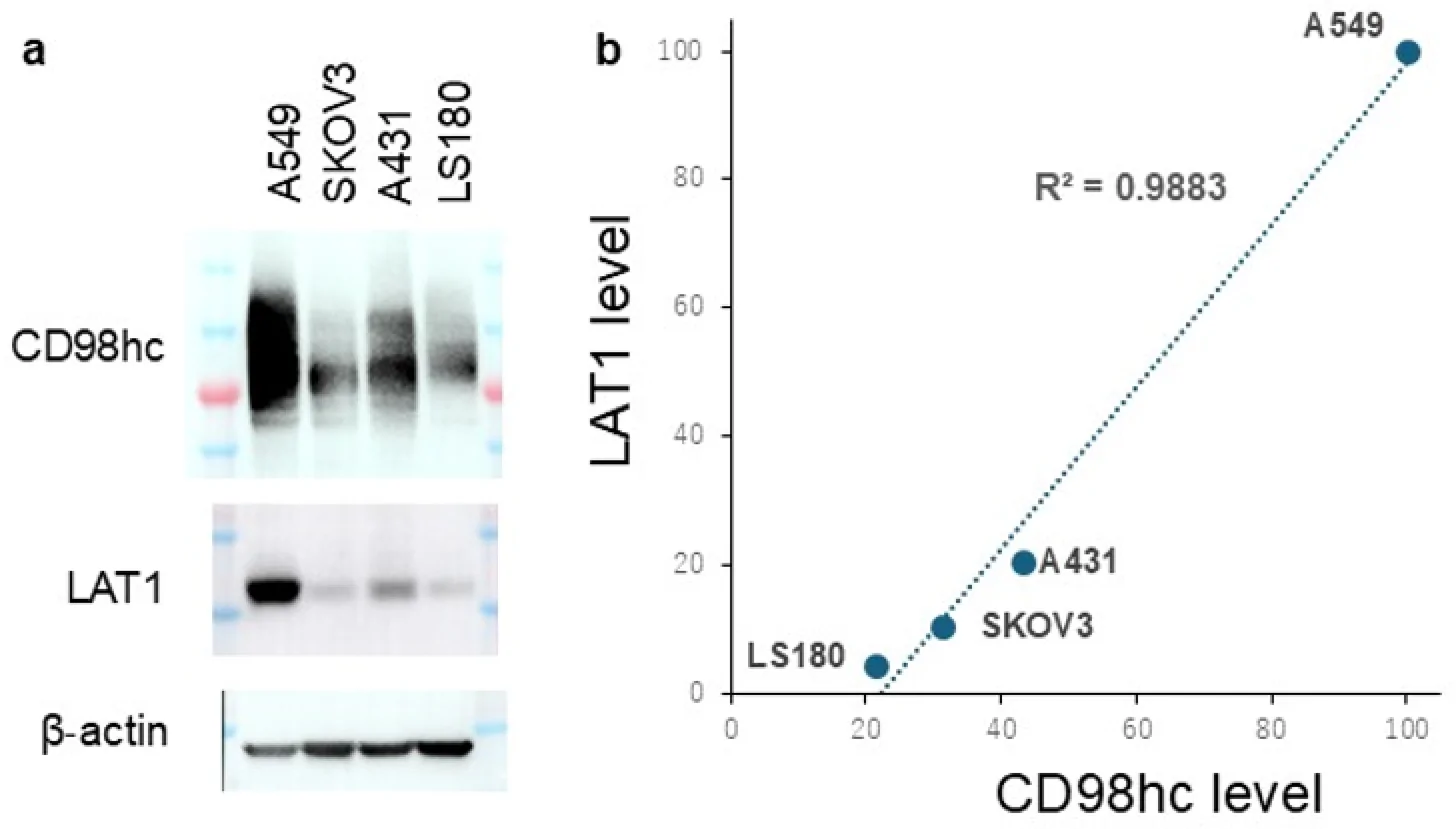
Expression of LAT-1 and CD98hc in the A549, SKOV3, A431, and LS18 cells lines. (**a**) Western blot analysis of CD98hc, LAT-1, and β-actin of total protein from the A549, SKOV3, A431, and LS180 cell lines. (**b**)The correlation of CD98hc levels with LAT-1 expression.

**Fig. 2:**
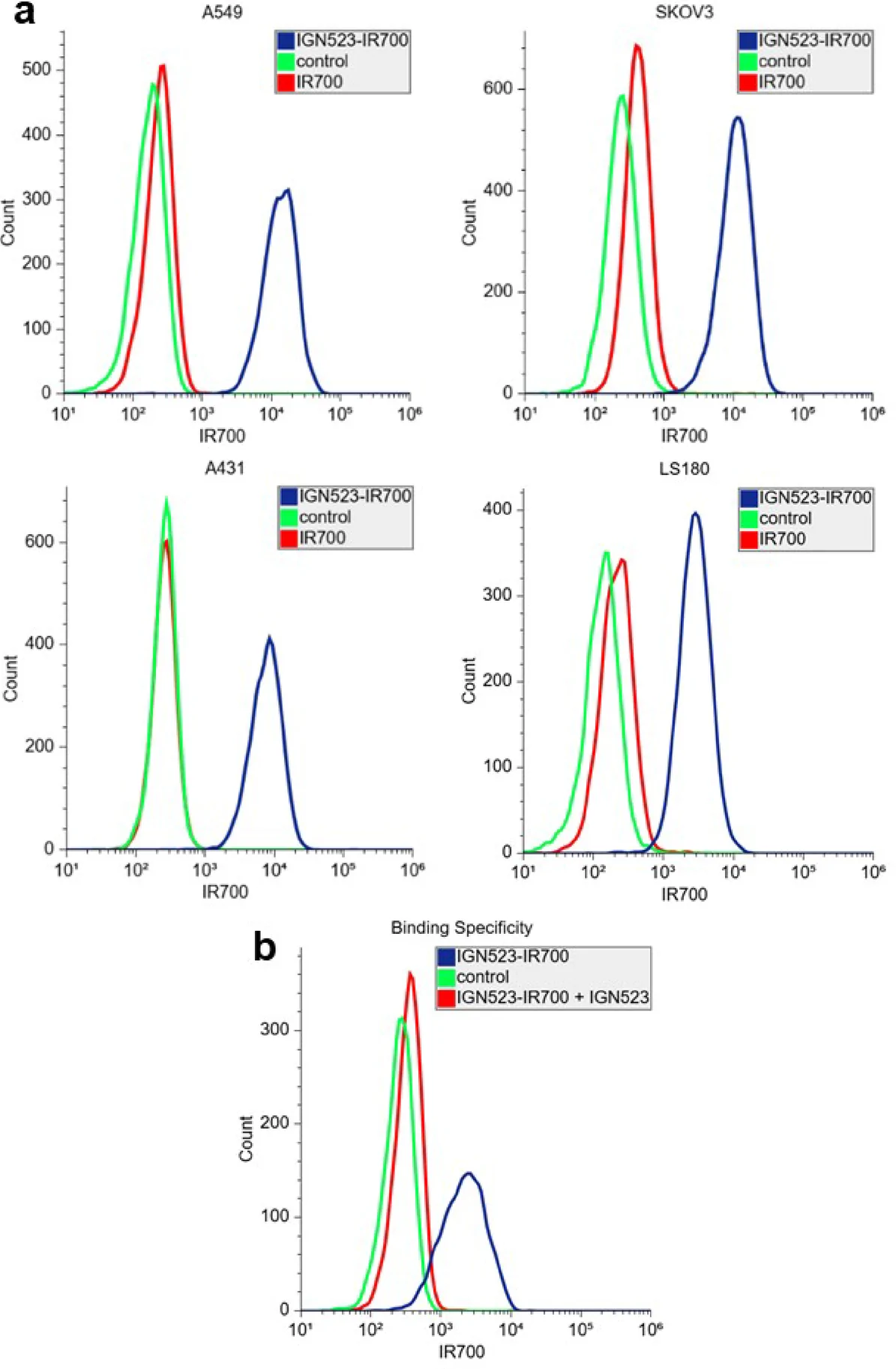
Binding and specific binding of IGN523-IR700 on the cells. (**a**) Binding was assessed in the A549, SKOV3, A431, and LS18 cell lines at 37 °C using flow cytometry. The cells were incubated with IR700 (red), IGN523-IR700 (blue), or phenol-red-free Roswell Park Memorial Institute 1640 medium (green, control). (**b**) Binding specificity was evaluated in A549 cells after 1 h of incubation at 4 °C. The IGN523-IR700 was almost completely inhibited with a 150-fold excess of unlabeled IGN523. The binding specificity of IGN523-IR700 was measured by flow cytometry.

Western blot analysis showed that all cell lines used in this study expressed both CD98hc and LAT1, with both proteins following the same rank order of expression: A549 > A431 > SKOV3 > LS180. Expression levels normalized to β-actin showed a linear correlation between CD98hc and LAT1 (R² = 0.99) (Fig. 1). Full-length blot images are provided in the Supporting Information (Fig. S3). Flow cytometric analysis showed that IGN523-IR700 bound to all tested cell lines, with staining indices of 83.4, 38.0, 37.9, and 12.5 for A549, A431, SKOV3, and LS180 cells, respectively (Fig. 2a and Tab. S1), following the same rank order as CD98hc expression determined by Western blot. Free IR700 alone did not show detectable cellular binding. Furthermore, co-incubation with excess unlabeled IGN523 resulted in a 92% reduction in the mean fluorescence intensity (MFI) of IGN523-IR700 in A549 cells, confirming the specificity of IGN523-IR700 binding to CD98hc (Fig. 2b and Table S1).

### In vitro NIR-PIT

**Fig. 3:**
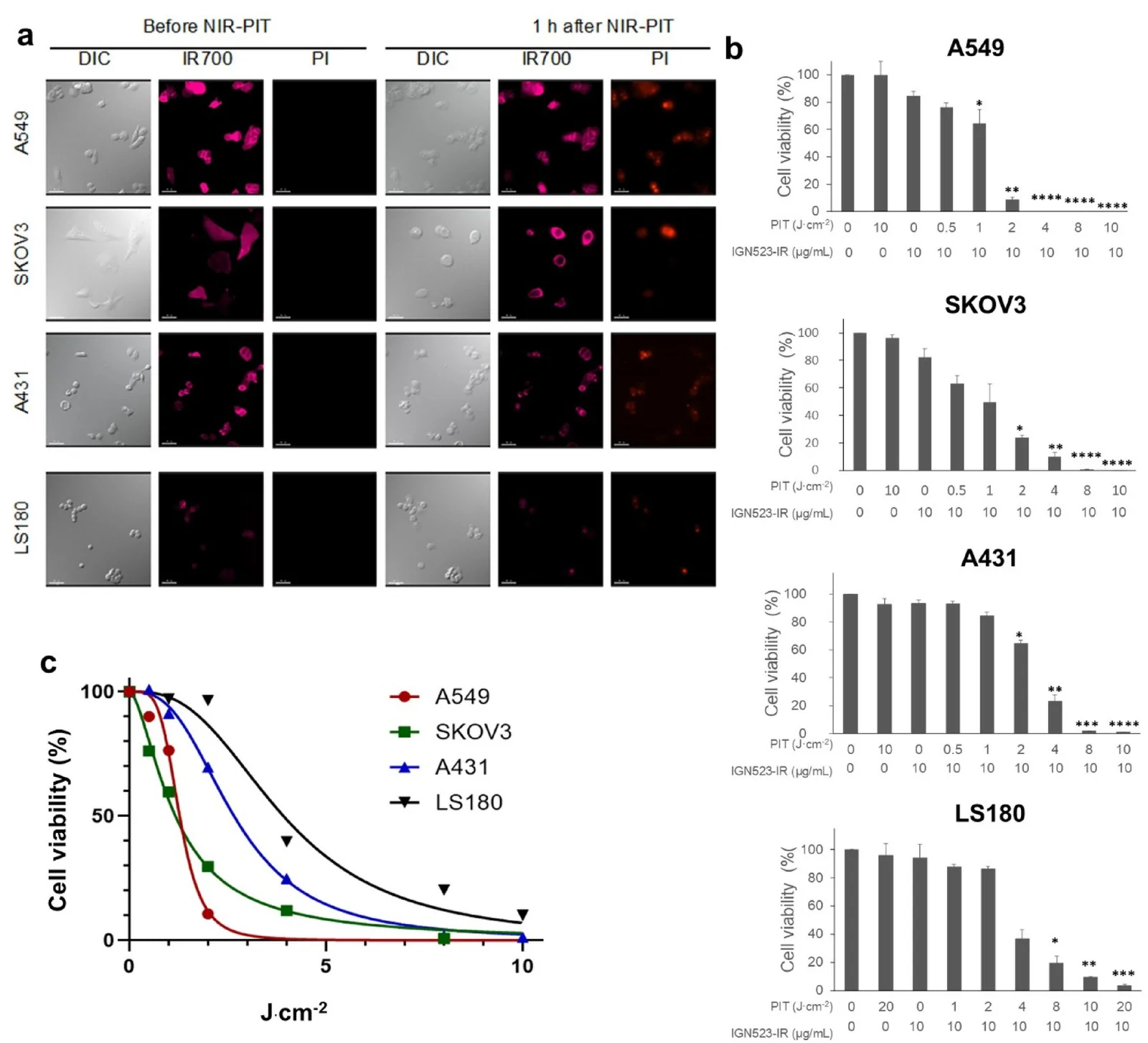
IGN523-IR700-mediated NIR-PIT in cell lines expressing CD98hc in vitro. (**a**) Microscopic observations of cells before and after exposure to 10 J·cm⁻² of NIR light with IGN523-IR700. Representative DIC and fluorescence images of IR700 and PI. Scale bar = 20 μm. (**b**) Cell viability was assessed using Cell Counting Kit-8 assays after treatment. (**c**) EC90 of light-dose PIT response in each cell line. Data are presented as mean ± standard error of the mean. Statistical differences were evaluated using Dunnett’s test (*p < 0.05; **p < 0.01; ***p < 0.001; ***p < 0.0001). DIC, differential interference contrast; NIR-PIT, near-infrared photoimmunotherapy; PI, propidium iodide.

One hour after NIR-PIT at 10 J·cm⁻², propidium iodide (PI) uptake and morphological changes characteristic of PIT, such as bleb formation and swelling, were observed in all cell lines (Fig. 3a). In LS180 cells, which exhibited low drug binding, morphological changes and PI uptake were less pronounced than in the other cell lines.

The cytotoxicity of NIR-PIT combined with IGN523-IR700 was evaluated 24 h after exposure to NIR light. Therapeutic efficacy was observed in all cell lines, and cell viability decreased in a light- dose-dependent manner (Fig. 3b). The light- dose required to achieve 90% cell death (EC₉₀) was 2.1, 4.5, 5.8, and 8.8 J·cm⁻² for the A549, SKOV3, A431, and LS180 cell lines, respectively (Fig. 3c). Cell viability in the light-only and drug-only groups was not significantly different from that of the control group across all cell lines. These results confirmed that neither NIR light nor IGN523-IR700 alone exerted therapeutic effects in the absence of other components.

### In vivo NIR-PIT

**Fig. 4:**
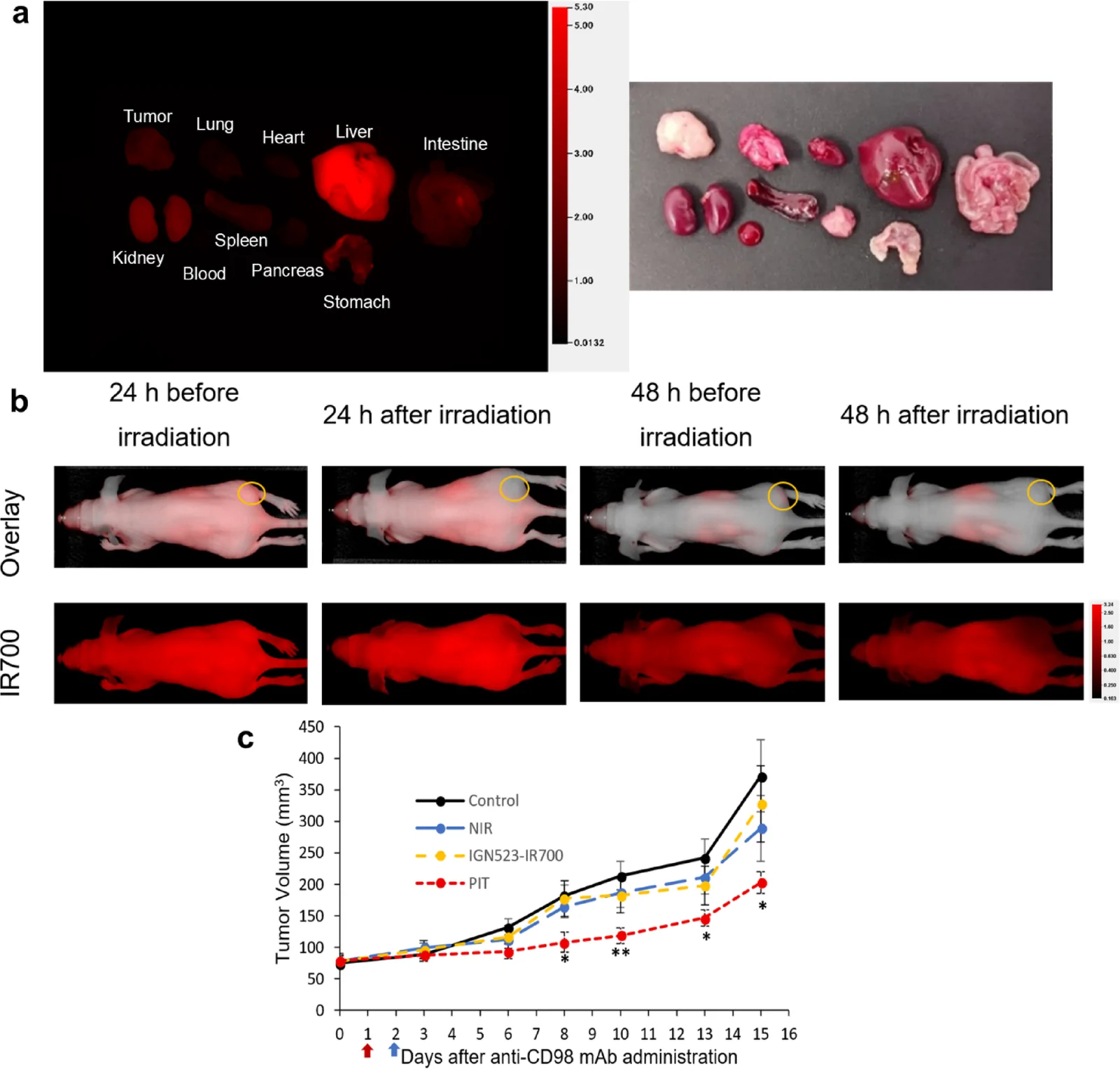
Evaluation of NIR-PIT in A549 tumor-bearing mice. (**a**) Fluorescence images of the A549 tumor and major organs 24 h after IGN523-IR700 injection. (**b**) Representative fluorescence images 24 h and 48 h after IGN523-IR700 injection, taken before and after NIR light irradiation in the NIR-PIT group. The yellow circles indicate the tumor location and the irradiation area. (**c**) Average tumor volume in each group. The red arrow indicates the time of 50 J·cm⁻² irradiation, and the blue arrow indicates 100 J·cm⁻² irradiation. Data are presented as mean ± standard error of the mean (*n* ≥ 6). Statistical differences were evaluated using Dunnett’s test (*p < 0.05; **p < 0.01). NIR-PIT, near-infrared photoimmunotherapy.

The biodistribution of IR700 fluorescence 24 h after the intravenous injection of IGN523-IR700 is shown in Fig. 4a. Fluorescence signals were detected in A549 tumors, although the highest fluorescence intensity was observed in the liver. Tumor-localized fluorescence diminished after tumor-directed NIR light irradiation. At 48 h post-injection (24 h after the first irradiation), fluorescence reappeared in the tumor region, and subsequent NIR light irradiation resulted in fluorescence reduction (Fig. 4b). Additional supporting semi-quantitative information is provided in the Tab. S3. Tumor growth was significantly inhibited in the NIR-PIT group compared with that in the untreated control group from day 8 onward (Fig. 4c, *p* < 0.05). Neither the drug-only nor the light-only groups showed significant differences in tumor growth compared with the untreated control group at any time point.

## Discussion

In this study, we investigated the potential of CD98hc as a novel target for NIR-PIT using IGN523, a humanized IgG1 antibody targeting CD98hc that has demonstrated a favorable safety profile in Phase I clinical trials^32^. We successfully prepared an IGN523-IR700 conjugate and demonstrated its therapeutic efficacy in vitro and in vivo.

CD98hc is a membrane protein expressed in most human organs and upregulated in many tumor types^17,19–25^. Therefore, it may serve as a potential therapeutic target for multiple cancer types. Although some amount of the drug will also accumulate to normal organs, with NIR-PIT, there may be little concern about side effects without light irradiation. In addition, as NIR-PIT involves a single drug administration per treatment session, even if the antibody itself has pharmacological effects, these effects are unlikely to be significant.

In this study, IGN523-IR700 bound to all four tested cancer cell lines. Even though the binding affinity of IGN523 was decreased by conjugating —of average of 3.62 IR700 molecules, the binding was sufficient to show a therapeutic effect. In addition, the binding of IGN523-IR700 to A549 cells, which highly express CD98hc, was inhibited by an excess of unlabeled IGN523, indicating that IGN523-IR700 maintains specific binding to CD98hc. Further consideration is needed to determine the appropriate number of IR700 bound to the antibody, and site-specific modifications, such as those used with antibody-drug conjugates, would likely be preferable. IGN523-IR700 exerted therapeutic effects upon NIR light irradiation, resulting in observable membrane damage, as evidenced by bleb formation and swelling. Therapeutic efficacy was light-dose-dependent and correlated with the staining index of cell-associated fluorescence quantified by flow cytometry. These observations suggest that the antitumor effects were consistent with the established mechanism of NIR-PIT. These findings indicate that CD98hc-targeted NIR-PIT has broad applications and the potential to expand applicability beyond the specific cancers evaluated in this study.

In the in vivo study, a significant reduction in tumor volume was observed in the NIR-PIT group compared to the control group beginning on day 8. Although tumor growth suppression remained statistically significant on day 15, tumor regrowth was observed in the treatment group. This phenomenon has been reported in many preclinical animal studies using immunodeficient mice, and EGFR-targeted antibodies that show excellent clinical outcomes exhibit tumor regrowth in animal experiments^33–35^.

In this study, the observation period was limited to 15 days to focus on the direct cytotoxic effects of NIR-PIT, as immune-mediated long-term tumor control cannot be adequately assessed in this model. NIR-PIT not only exerts direct cytotoxic effects, but also triggers immunogenic cell death and subsequent antitumor immune responses. Therefore, the absence of intact immunity in nude mice may alter the observed therapeutic response. Many studies have been conducted on the immune responses induced by NIR-PIT using a syngeneic mouse model^36^. Future studies using immunocompetent models would enable the long-term evaluation of the immune-mediated component of CD98hc-targeted NIR-PIT.

In addition, the use of a murine model imposes limitations on in vivo pharmacokinetic interpretations. IGN523 specifically targets human CD98hc and does not interact with murine CD98hc, as evidenced by the absence of antitumor activity against the mouse melanoma cell line B16-F10^37^. Therefore, the biodistribution observed in this study does not reflect the influence of endogenous CD98hc expressed in normal tissues. Although IGN523-IR700 exhibited a biodistribution profile broadly consistent with that of other antibody–dye conjugates^2,38^, its pharmacokinetic behavior in humans may be substantially affected by binding to CD98hc in normal tissues. Thus, the pharmacological effects of IGN523 on normal CD98hc-expressing human tissues should be carefully investigated in future translational studies.

An important aspect of CD98hc-targeted therapy is its potential integration with LAT1-targeted nuclear imaging within a theranostic framework. LAT1 forms a functional heterodimer with CD98hc at the cell membrane, facilitating amino acid transport into cancer cells, and is overexpressed in many tumor types, with expression levels correlated with malignancy^14,39–41^. Therefore, CD98hc expression levels were expected to correlate with those of LAT1^26–28^, a relationship supported by our western blot analysis, which showed that both proteins followed the same rank order across the four tested cell lines (A549 > A431 > SKOV3 > LS180). A strong correlation between CD98hc and LAT1 expression has also been reported in breast cancer cell lines and patient samples^42^. LAT1-targeted PET imaging has been validated and is clinically available for various cancers, providing a highly specific method for visualizing malignant tissues with minimal background signal in normal tissues^14^. In addition, using the developed LAT1-targeted PET tracer, A549 and LS180 tumors used in this study were clearly visualized in murine xenografts^43^. Based on this association, we hypothesize that patients with LAT1-PET-positive tumors represent a population with high CD98hc expression on tumor cell surfaces and may therefore be candidates for CD98hc-targeted NIR-PIT. This theranostic integration remains a future goal that warrants validation in in vivo models. However, combining CD98hc-targeted therapy with LAT1 imaging may ultimately enhance the precision, effectiveness, and safety of cancer treatments, supporting personalized approaches in oncology.

This study provides preclinical evidence that NIR-PIT is effective against CD98hc-positive tumors both in vitro and in vivo, establishing the feasibility of CD98hc-targeted NIR-PIT. To further advance the proposed CD98hc/LAT1-targeted PIT/PET theranostic strategy, several issues must be addressed in future studies. First, the treatment protocol, including the drug dose, irradiation timing, and number of irradiation sessions, was modeled on previously reported NIR-PIT studies and was not optimized. Repeated irradiation has been commonly used in preclinical NIR-PIT studies because the first irradiation induces extensive cell death, particularly in perivascular tumor cells, thereby increasing tumor vascular permeability and facilitating re-accumulation of the conjugate in the residual tumor tissue. Re-accumulation of IGN523-IR700 was also observed in our experiments, suggesting that the number and timing of irradiation sessions warrant further optimization. Therefore, a comprehensive pharmacokinetic characterization of IGN523-IR700 and systematic optimization of these treatment parameters are considered primary priorities for future work. With respect to on-target/off-tumor effects in normal tissues, the use of antibodies that selectively recognize the CD98hc/LAT1 complex—such as recently developed complex-specific antibodies—may represent a promising strategy for improving tumor selectivity. Second, the in vivo study used only A549 xenografts, which showed the highest CD98hc expression among the tested cell lines. To validate the theranostic concept, future studies should assess the correlation between LAT1/CD98hc expression levels and LAT1-PET imaging findings, evaluate NIR-PIT therapeutic efficacy across tumor models with varying LAT1/CD98hc expression levels, and address the limitations of the nude mouse model by developing more appropriate animal models.

In conclusion, we demonstrated the therapeutic efficacy of CD98hc-targeted NIR-PIT in vitro and in vivo, establishing CD98hc as a viable target for NIR-PIT. Targeting CD98hc, which is highly expressed in various tumors, may provide a foundation for expanding NIR-PIT applications to diverse cancer types. CD98hc-targeted therapy holds promise for integration with LAT1 imaging, potentially contributing to precision medicine and personalized treatment strategies for future NIR-PIT applications.

## Materials and methods

### Antibodies and reagents

IR700 NHS ester was purchased from EOS Med Chem (Jinan City, China). Humanized anti-CD98hc (IGN523) monoclonal antibody was purchased from Selleck (Houston, TX, USA). All other chemicals were reagent grade.

### Synthesis of IGN523-IR700 and IGN523-FITC

IGN523 (3 mg·mL⁻¹, 20 nmol) was incubated overnight with IR700 NHS ester (10 mM, 160 nmol) in 0.1 mol·L⁻¹ Na₂HPO₄ (pH 8.5) at room temperature. For IGN523-FITC synthesis, 3 mg·mL⁻¹ (6.7 nmol) of IGN523 was mixed with FITC NHS ester (1 mM, 13.4 nmol) under the same conditions. The reaction mixture was purified with a Sephadex G25 column (PD-10; GE Healthcare, Piscataway, NJ, USA).

The purity of IGN523-IR700 was assessed by size-exclusion HPLC using a TOSOH TSKgel superSW 2000 column (4.6 D × 30 cm) with 0.1 M phosphate buffer (pH 6.7) at a flow rate of 0.3 mL/min. Protein concentration was determined using the BCA protein assay reagent (Fujifilm Wako Pure Chemical Industries, Osaka, Japan), and absorbance was measured at 562 nm using a spectrophotometer (V-730 Bio; JASCO, Tokyo, Japan). The concentrations of IR700 and FITC were determined spectrophotometrically by absorbance measurements at 689 nm and 495 nm, respectively, using IR700 (50 µM) and FITC (40 µM) as standards. The average degree of labeling (DoL) was calculated as the molar ratio of dye to antibody using the following formula:

$$\left[ {{\mathrm{Dye}}} \right]=\left( {{\text{absorbance of sample}} \cdot {\text{absorbance of standar}}{{\mathrm{d}}^{ - 1}}} \right) \cdot \left[ {{\mathrm{standard}}} \right]$$

$${\mathrm{DoL}}=\left[ {{\mathrm{Dye}}} \right] \cdot {\left[ {{\mathrm{IGN523}}} \right]^{ - 1}}$$

### Cell culture

The A549 (human lung carcinoma) and A431 (human epidermoid carcinoma) cell lines were purchased from RIKEN BRC (Tsukuba, Japan). The SKOV3 (human ovarian adenocarcinoma) and LS180 (human colorectal adenocarcinoma) cell lines were purchased from the American Type Culture Collection (Manassas, VA, USA). The cells were grown in Dulbecco’s modified Eagle’s medium (Fujifilm Wako Pure Chemical Industries) supplemented with 10% heat-inactivated fetal bovine serum and 1% penicillin–streptomycin (Wako Pure Chemical Industries) in tissue culture dishes. The cells were maintained at 37 °C in a humidified atmosphere containing 5% CO₂.

### LAT-1 and CD98hc expression

Cultured cells were lysed in Cell Lysis Buffer (Nacalai Tesque, Kyoto, Japan), and lysates were centrifuged to remove debris. Samples were diluted in Sample Buffer Solution with Reducing Reagent for SDS-PAGE (Nacalai Tesque) and loaded (10 µL, 2.0 mg·mL⁻¹ total protein) onto gels. Western blotting was performed using the following primary antibodies: anti-LAT1 (1:1000; KE026, Trans Genic Inc., Tokyo, Japan) and anti-CD98hc (1:1000; EPR27110-42, Abcam, Cambridge, UK). Horseradish peroxidase-conjugated secondary antibody (1:5000; #7076, Cell Signaling Technology, Danvers, MA, USA) was used for detection. β-Actin was used as a loading control and detected with anti-β-actin antibody (1:5000; NB600-505SS, Novus Biologicals, Littleton, CO, USA) and horseradish peroxidase-conjugated secondary antibody (1:5000; #7074, Cell Signaling Technology). Immunoreactive bands were visualized using Chemi-Lumi One L (Nacalai Tesque) and imaged with a FUSION SOLO.7 S.EDGE system (Vilber Bio Imaging, Marne-la-Vallée, France). A FastGene Bluestar prestained protein marker (NIPPON Genetics, Tokyo, Japan) was used to estimate molecular weights. Band intensities were quantified using the instrument’s built-in software, and LAT1 and CD98hc expression levels were normalized to β-actin for comparison.

### In vitro cell binding and specificity using flow cytometry

Flow cytometry analysis was performed to evaluate the binding of IGN523-IR700 to the A549, SKOV3, A431, and LS180 cells. A total of 2 × 10⁵ cells per sample were seeded into flow cytometry tubes and washed with cold PBS. Samples were incubated for 1 h in the dark at 37 °C with 200 µL of IR700 (278.8 nM) or IGN523-IR700 (69.7 nM). After incubation, the cells were washed with cold PBS to remove unbound conjugates, then resuspended in PBS.

The binding of IGN523-IR700 was compared with that of IGN523-FITC, the representative parent IGN523. A total of 2 × 10⁵ cells of A549 were added with varying amounts of antibody conjugates (0.001, 0.01, 0.1, 0.3, 1, 3, 10, and 30 µg). Fluorescence signals corresponding to IR700 and FITC were measured using a *BD FACSymphony*™ *A1*. Debris and doublets were excluded by forward- and side-scatter gating. Flow cytometry data were exported and combined for analysis using Floreada.io software. The staining index was calculated as follows: (mean fluorescence intensity (MFI) of sample – mean fluorescence intensity of control) / (SD of control × 2)^44^.

To validate the binding specificity, a total of 1 × 10⁵ A549 cells in 100 µL per sample were incubated for 1 h at 4 °C in the dark with PBS alone, IGN523-IR700 (69.7 nM), or IGN523-IR700 (69.7 nM) in the presence of a 150-fold molar excess of unlabeled IGN523. The percent of inhibition was calculated as 1–(MFI of IGN523-IR700 + IGN523 sample / MFI of IGN523-IR700 sample) × 100%.

### In vitro microscopic analysis

A549, SKOV3, A431, and LS180 cells were seeded at a density of 5.5 × 10⁴ cells per well on glass-bottom dishes and incubated for 24 h. Cells were then incubated with 2 mL of fresh culture medium containing 10 µg·mL⁻¹ IGN523-IR700 for 1 h at 37 °C. After washing with phosphate-buffered saline (PBS) (Fujifilm Wako Pure Chemical Industries), the cells were transferred to medium containing PI for double staining. Morphological changes were observed under an IX-83 inverted microscope (Olympus, Tokyo, Japan). Transmitted-light differential interference contrast (DIC) images were acquired. PI and IR700 fluorescence were detected using RFP (561 nm), and Cy5.5 (640 nm) filter sets (Olympus, Tokyo, Japan). NIR light was applied at 10 J·cm⁻² using a 690 nm excitation filter (Semrock, Rochester, NY, USA) attached to the IX-83 microscope at an irradiance of 55.6 mW·cm⁻² for 180 s. DIC and fluorescence images were acquired 1 h after NIR irradiation.

### In vitro NIR-PIT and cell viability assay

A549, SKOV3, A431, and LS180 cells were seeded into 24-well plates and incubated for 24 h. After washing with PBS, the cells were incubated with 300 µL of phenol-red-free Roswell Park Memorial Institute 1640 medium (Fujifilm Wako Pure Chemical Industries) containing 10 µg·mL⁻¹ IGN523-IR700 for 1 h at 37 °C. NIR light from light-emitting diodes at a wavelength of 690 nm with power 50 mW·cm⁻² (L690-66-60; Ushio-Epitex, Kyoto, Japan) was applied to the A549, SKOV3, A431 cells at actual doses up to 10 J·cm⁻² and to the LS180 cells up to 20 J·cm⁻². The actual power density was measured using an optical power meter (PM100; Thorlabs, Newton, NJ, USA). Following irradiation, the cells were incubated in fresh complete medium for 24 h. Cell viability after treatment was assessed using the Cell Counting Kit-8 assay (Dojindo, Kumamoto, Japan). The absorbance was measured at 450 nm using a GloMax Discover microplate reader (Promega, Madison, WI, USA). Cell viability curves were generated using GraphPad Prism, and EC_90_ values were calculated.

### In vivo NIR-PIT

All animal experiments were conducted in accordance with institutional guidelines and approved by the Institutional Animal Care Committee (protocol code: 25–055). This study is reported in accordance with ARRIVE guidelines. The mice were housed per group of 2–5 in standard laboratory conditions with a 12-h light/dark cycle, controlled temperature (21–23 °C), and humidity (40–60%). A minimum of six mice per group was used in all experiments. A549 cells (5 × 10⁶ cells in 100 µL) were suspended in a 1:1 (v/v) mixture of PBS and Matrigel (Corning Life Sciences, Corning, NY, USA). The cell suspension was injected subcutaneously into 6-week-old female BALB/c nu/nu mice with body weights ranging from 16 to 19 g (Jackson Laboratory Japan, Yokohama, Japan).

In vivo studies were initiated when tumor volumes exceeded 50 mm³. The average tumor volume of A549 xenografts at the start of therapeutic studies was 77.7 ± 7.6 mm³. Tumor volume was measured using an external caliper and calculated using the formula: volume (mm³) = length × width² × 0.5.

The mice were randomly assigned to four groups: (1) no treatment (control), (2) NIR light irradiation without IGN523-IR700 (light-only), (3) IGN523-IR700 (100 µL of 1 mg·mL⁻¹ IGN523-IR700 per mouse) without light irradiation (drug-only), and (4) IGN523-IR700 followed by NIR light irradiation (NIR-PIT). IGN523-IR700 was intravenously injected with 100 µL of 1 mg·mL⁻¹ IGN523-IR700 per mouse. NIR light exposure (50 J·cm⁻² on day 1 and 100 J·cm⁻² on day 2 after IGN523-IR700 injection) was performed using an FD1 frontal light diffuser (Medlight, Ecublens, Switzerland) equipped with an ML7710 laser (diameter spot size 1 cm and power 150 mW·cm⁻²). Fluorescence imaging of IR700 before and after NIR light exposure was conducted using a Pearl Trilogy small-animal imaging system (LI-COR Biosciences, Lincoln, NE, USA) 24 h and 48 h after IGN523-IR700 injection.

For ex vivo imaging studies, a mouse was prepared as described previously and euthanized 24 h after IGN523-IR700 injection. Then, the tumor and organs were excised and imaged using a Pearl Trilogy small-animal imaging system.

### Humane endpoints and animal welfare

Mice were monitored daily to evaluate their overall health and behavior, specifically for indications of distress. If any subjects displayed marked distress or a decline in their condition, euthanasia was administered. Intervention criteria included symptoms such as abnormal posture, social withdrawal, or tumor volume exceeding 1500 mm³. Euthanasia procedures were conducted using inhalant anesthesia (isoflurane, 2% for more than 10 min), followed by cervical dislocation, carried out in a designated room to reduce stress on the animals. Continuous monitoring for signs of distress was implemented throughout the procedure.

### Statistical analysis

All results are expressed as mean ± standard error of the mean. Statistical analyses were performed using Microsoft Excel (Microsoft Corp., Redmond, WA, USA). Comparisons among groups were performed using Dunnett’s test. The minimum level of significance was set at *p* < 0.05.

## Supplementary Information

Below is the link to the electronic supplementary material.

Supplementary Material 1

## Data Availability

All data generated or analysed during this study are included in this published article and its supplementary information files.

## References

[1] Kobayashi, H. & Choyke, P. L. Near-infrared photoimmunotherapy of cancer. *Acc. Chem. Res.* **52**, 2332–2339 (2019). 31335117 10.1021/acs.accounts.9b00273 PMC6704485

[2] Sato, K. et al. Photoinduced ligand release from a silicon phthalocyanine dye conjugated with monoclonal antibodies: A mechanism of cancer cell cytotoxicity after near-infrared photoimmunotherapy. *ACS Cent. Sci.* **4**, 1559–1569 (2018). 30555909 10.1021/acscentsci.8b00565 PMC6276043

[3] Mitsunaga, M. et al. Cancer cell–selective in vivo near infrared photoimmunotherapy targeting specific membrane molecules. *Nat. Med.* **17**, 1685–1691 (2011). 22057348 10.1038/nm.2554 PMC3233641

[4] Nagaya, T. et al. Near-infrared photoimmunotherapy targeting prostate cancer with prostate-specific membrane antigen (PSMA) antibody. *Mol. Cancer Res.* **15**, 1153–1162 (2017). 28588059 10.1158/1541-7786.MCR-17-0164 PMC5581714

[5] Maawy, A. A. et al. Near infra-red photoimmunotherapy with anti-CEA-IR700 results in extensive tumor lysis and a significant decrease in tumor burden in orthotopic mouse models of pancreatic cancer. *PLoS One*. **10**, e0121989 (2015). 25799218 10.1371/journal.pone.0121989 PMC4370703

[6] Hanaoka, H. et al. Glypican-3 targeted human heavy chain antibody as a drug carrier for hepatocellular carcinoma therapy. *Mol. Pharm.* **12**, 2151–2157 (2015). 25955255 10.1021/acs.molpharmaceut.5b00132 PMC7720675

[7] Nagaya, T. et al. Near infrared photoimmunotherapy with an anti-mesothelin antibody. *Oncotarget* **7**, 23361–23369 (2016). 26981775 10.18632/oncotarget.8025 PMC5029632

[8] Nakajima, T., Sano, K., Choyke, P. L. & Kobayashi, H. Improving the efficacy of photoimmunotherapy (PIT) using a cocktail of antibody conjugates in a multiple antigen tumor model. *Theranostics* **3**, 357–365 (2013). 23781283 10.7150/thno.5908 PMC3677407

[9] Nagaya, T. et al. Near infrared photoimmunotherapy of B-cell lymphoma. *Mol. Oncol.* **10**, 1404–1414 (2016). 27511870 10.1016/j.molonc.2016.07.010 PMC5067212

[10] Nagaya, T. et al. Near infrared photoimmunotherapy with avelumab, an anti-programmed death-ligand 1 (PD-L1) antibody. *Oncotarget* **8**, 8807–8817 (2017). 27716622 10.18632/oncotarget.12410 PMC5341755

[11] Nagaya, T. et al. Syngeneic mouse models of oral cancer are effectively targeted by anti–CD44-based NIR-PIT. *Mol. Cancer Res.* **15**, 1667–1677 (2017). 28923838 10.1158/1541-7786.MCR-17-0333 PMC5712275

[12] Jin, J., Krishnamachary, B., Mironchik, Y., Kobayashi, H. & Bhujwalla, Z. M. Phototheranostics of CD44-positive cell populations in triple negative breast cancer. *Sci. Rep.* **6**, 27871 (2016). 27302409 10.1038/srep27871 PMC4908597

[13] Jing, H. et al. Imaging and selective elimination of glioblastoma stem cells with theranostic near-infrared-labeled CD133-specific antibodies. *Theranostics* **6**, 862–874 (2016). 27162556 10.7150/thno.12890 PMC4860894

[14] Achmad, A. et al. LAT1-specific PET radiotracers: Development and clinical experiences of a new class of cancer-specific radiopharmaceuticals. *Theranostics* **15**, 1864–1878 (2025). 39897549 10.7150/thno.99490 PMC11780518

[15] Wei, L. et al. Specific transport of 3-fluoro‐l‐α‐methyl‐tyrosine by LAT1 explains its specificity to malignant tumors in imaging. *Cancer Sci.* **107**, 347–352 (2016). 26749017 10.1111/cas.12878 PMC4814262

[16] Minoji, T. et al. The clinical potential of FBPA-PET/CT imaging for colorectal cancer and its liver metastases expressing LAT1. *Anticancer Res.* **43**, 3913–3921 (2023). 37648310 10.21873/anticanres.16579

[17] Xia, P. & Dubrovska, A. CD98 heavy chain as a prognostic biomarker and target for cancer treatment. *Front. Oncol.* **13**, 1251100 (2023). 37823053 10.3389/fonc.2023.1251100 PMC10562705

[18] Furuya, M., Horiguchi, J., Nakajima, H., Kanai, Y. & Oyama, T. Correlation of L-type amino acid transporter 1 and CD98 expression with triple negative breast cancer prognosis. *Cancer Sci.* **103**, 382–389 (2012). 22077314 10.1111/j.1349-7006.2011.02151.x

[19] Köseer, A. S. et al. Validation of CD98hc as a therapeutic target for a combination of radiation and immunotherapies in head and neck squamous cell carcinoma. *Cancers (Basel)*. **14**, 1677 (2022). 35406454 10.3390/cancers14071677 PMC8997111

[20] Haining, Z. et al. Relation of LAT1/4F2hc expression with pathological grade, proliferation and angiogenesis in human gliomas. *BMC Clin. Pathol.* **12**, 4 (2012). 22373026 10.1186/1472-6890-12-4 PMC3305678

[21] Lee, D. et al. Expression profile of CD98 heavy chain and L-type amino acid transporter 1 and its prognostic significance in colorectal cancer. *Pathol. Res. Pract.* **229**, 153730 (2022). 34942513 10.1016/j.prp.2021.153730

[22] Bianconi, D. et al. Expression of CD98hc in pancreatic cancer and its role in cancer cell behavior. *J. Cancer*. **13**, 2271–2280 (2022). 35517419 10.7150/jca.70500 PMC9066202

[23] Yaga, M. et al. CD98 heavy chain protein is overexpressed in non-small cell lung cancer and is a potential target for CAR T-cell therapy. *Sci. Rep.* **14**, 17917 (2024). 39095551 10.1038/s41598-024-68779-9 PMC11297167

[24] Ichinoe, M. et al. Prognostic values of L-type amino acid transporter 1 and CD98hc expression in breast cancer. *J. Clin. Pathol.* **74**, 589–595 (2021). 32907912 10.1136/jclinpath-2020-206457 PMC8380907

[25] Edavettal, S. et al. Enhanced delivery of antibodies across the blood-brain barrier via TEMs with inherent receptor-mediated phagocytosis. *Med* **3**, 860–882e15 (2022). 36257298 10.1016/j.medj.2022.09.007

[26] Satoh, T. et al. Prognostic significance of the expression of CD98 (4F2hc) in gastric cancer. *Anticancer Res.* **37**, 631–636 (2017). 28179310 10.21873/anticanres.11357

[27] Kaira, K. et al. Relationship between LAT1 expression and response to platinum-based chemotherapy in non-small cell lung cancer patients with postoperative recurrence. *Anticancer Res.* **31**, 3775–3782 (2011). 22110199

[28] Kaira, K. et al. Prognostic significance of L-type amino-acid transporter 1 expression in surgically resected pancreatic cancer. *Br. J. Cancer*. **107**, 632–638 (2012). 22805328 10.1038/bjc.2012.310 PMC3419959

[29] Isoda, A. et al. Expression of L-type amino acid transporter 1 (LAT1) as a prognostic and therapeutic indicator in multiple myeloma. *Cancer Sci.* **105**, 1496–1502 (2014). 25220100 10.1111/cas.12529 PMC4462375

[30] Yanagisawa, N. et al. High expression of L-type amino acid transporter 1 as a prognostic marker in bile duct adenocarcinomas. *Cancer Med.* **3**, 1246–1255 (2014). 24890221 10.1002/cam4.272 PMC4302674

[31] Yazawa, T. et al. Clinical significance of coexpression of L-type amino acid transporter 1 (LAT1) and ASC amino acid transporter 2 (ASCT2) in lung adenocarcinoma. *Am. J. Transl Res.* **7**, 1126–1139 (2015). 26279756 PMC4532745

[32] Bixby, D. et al. A phase I study of IGN523, a novel anti-CD98 monoclonal antibody in patients with relapsed or refractory acute myeloid leukemia (AML). *Blood* **126**, 3809–3809 (2015).

[33] Siddiqui, M. R. et al. Targeting epidermal growth factor receptor (EGFR) and human epidermal growth factor receptor 2 (HER2) expressing bladder cancer using combination photoimmunotherapy (PIT). *Sci. Rep.* 9, (2019). (2084). 10.1038/s41598-019-38575-x PMC6375935 30765854

[34] Tian, J., Chen, C., Du, X. & Wang, M. Near-infrared photoimmunotherapy in cancer treatment: a bibliometric and visual analysis. *Front. Pharmacol.* **15**, 1485242 (2024). 39498336 10.3389/fphar.2024.1485242 PMC11533137

[35] Wang, X., Luo, D. & Basilion, J. P. Photodynamic therapy: Targeting cancer biomarkers for the treatment of cancers. *Cancers (Basel)*. **13**, 2992 (2021). 34203805 10.3390/cancers13122992 PMC8232794

[36] Fukushima, H. et al. Antitumor host immunity enhanced by near-infrared photoimmunotherapy. *Cancer Sci.* **116**, 572–580 (2025). 39663860 10.1111/cas.16427 PMC11875768

[37] Hayes, G. M. et al. Antitumor activity of an anti-CD98 antibody. *Int. J. Cancer*. **137**, 710–720 (2015). 25556716 10.1002/ijc.29415 PMC6680144

[38] Yan, Y., Vasudevan, S., Nguyen, H. T. T. & Merlin, D. Intestinal epithelial CD98: An oligomeric and multifunctional protein. *Biochim. Biophys. Acta*. **1780**, 1087–1092 (2008). 18625289 10.1016/j.bbagen.2008.06.007 PMC2602860

[39] Lee, Y. et al. Cryo-EM structure of the human L-type amino acid transporter 1 in complex with glycoprotein CD98hc. *Nat. Struct. Mol. Biol.* **26**, 510–517 (2019). 31160781 10.1038/s41594-019-0237-7

[40] Xia, P. CD98 light chain LAT1 tracers in PET-CT diagnosis of cancer patients. *Anticancer Agents Med. Chem* **25**, (2025). 10.2174/0118715206381956250622145301 40611417

[41] Napolitano, L. et al. LAT1 is the transport competent unit of the LAT1/CD98 heterodimeric amino acid transporter. *Int. J. Biochem. Cell. Biol.* **67**, 25–33 (2015). 26256001 10.1016/j.biocel.2015.08.004

[42] Montero, J. C. et al. Surfaceome analyses uncover CD98hc as an antibody drug-conjugate target in triple negative breast cancer. *J. Exp. Clin. Cancer Res.* **41**, 106 (2022). 10.1186/s13046-022-02330-4 PMC8941813 35317825

[43] Hanaoka, H. et al. Novel 18F-labeled α-methyl-phenylalanine derivative with high tumor accumulation and ideal pharmacokinetics for tumor-specific imaging. *Mol. Pharm.* **16**, 3609–3616 (2019). 31242385 10.1021/acs.molpharmaceut.9b00446

[44] Kalina, T. et al. EuroFlow standardization of flow cytometer instrument settings and immunophenotyping protocols. *Leukemia* **26**, 1986–2010 (2012). 22948490 10.1038/leu.2012.122 PMC3437409

