# Supplementary material for: Therapeutic potential of photoimmunotherapy in solid tumors expressing CD98 heavy chain
Source: Sci Rep. 2026 Jun 9;16:26141. doi: 10.1038/s41598-026-57005-3 (PMC13490449; doi:10.1038/s41598-026-57005-3)
Supplement: Supplementary file 1 [file 41598_2026_57005_MOESM1_ESM.docx]

**Supplementary**

**Figure S1.** A chromatogram of IGN523-IR700 at 700 nm. The purity of antibody conjugates was evaluated by HPLC.

**Figure S2. The binding of IGN523-IR700 and IGN523-FITC *in vitro*.** The binding of IGN523-IR700 and IGN523-FITC to A549 in serial amounts of antibody-labeled.

**Figure S3**. Western blot analysis of CD98, LAT-1, and β-actin from A549, SKOV3, A431, and LS180 cell lines.

**Table S1**. Raw data for Figure 2. The mean fluorescence intensity was analyzed from flow cytometry data using Floreada.io.

| **Cell Line** | **Group** | **Mean fluorescence intensity**  **(MFI)** | **Stain Index** |
| --- | --- | --- | --- |
| Cell binding |  |  |  |
| A549 | control | 191.42 |  |
|  | SD of control | 93.48 |  |
|  | IR700 | 268.05 | 0.41 |
|  | IGN523-IR700 | 15781.15 | 83.39 |
| SKOV3 | control | 279.15 |  |
|  | SD of control | 161.59 |  |
|  | IR700 | 481.07 | 0.62 |
|  | IGN523-IR700 | 12512.37 | 37.85 |
| A431 | control | 287.75 |  |
|  | SD of control | 113.81 |  |
|  | IR700 | 285.00 | -0.01 |
|  | IGN523-IR700 | 8943.41 | 38.03 |
| LS180 | control | 169.18 |  |
|  | SD of control | 134.40 |  |
|  | IR700 | 269.64 | 0.37 |
|  | IGN523-IR700 | 3439.73 | 12.17 |
| Binding specificity |  |  |  |
| A549 | control | 283.64 |  |
|  | SD of control | 129.88 |  |
|  | IGN523-IR700+IGN523 | 469.82 | 0.72 |
|  | IGN523-IR700 | 2713.79 | 9.36 |
|  | Percent of inhibition | 92.31% |  |

**Table S2**. Raw data for Figure 3b. Cell viability was assessed by Cell Counting Kit-8 assays after treatment. Each value represents the measurement result for each well (n = 3).

| **Cell viability %** | | | | | | | | | | |
| --- | --- | --- | --- | --- | --- | --- | --- | --- | --- | --- |
| **PIT (J·cm⁻²)** | | **0** | **10** | **0** | **0.5** | **1** | **2** | **4** | **8** | **10** |
| **IGN523-IR (μg·mL⁻¹)** | | **0** | **10** | **10** | **10** | **10** | **10** | **10** | **10** | **10** |
| **A549** |  | 100.00 | 102.06 | 83.90 | 74.12 | 72.15 | 9.89 | -0.29 | -0.67 | -0.79 |
|  |  |  | 88.77 | 82.96 | 79.60 | 68.66 | 9.66 | 0.10 | -0.21 | -0.34 |
|  |  |  | 108.64 | 88.36 | 75.82 | 53.17 | 7.40 | 0.17 | -0.04 | -0.28 |
|  | **average** | **100.00** | **99.82** | **85.07** | **76.51** | **64.66** | **8.98** | **-0.01** | **-0.31** | **-0.47** |
|  | **SEM** |  | **10.12** | **2.88** | **2.80** | **10.10** | **1.37** | **0.25** | **0.33** | **0.28** |
| **SKOV3** |  | 100.00 | 91.32 | 71.59 | 50.96 | 46.02 | 26.79 | 4.33 | 0.34 | -1.52 |
|  |  |  | 98.70 | 82.70 | 67.06 | 28.80 | 21.37 | 14.98 | 0.56 | -0.27 |
|  |  |  | 98.74 | 93.18 | 70.88 | 74.23 | 23.77 | 10.87 | 1.11 | 0.16 |
|  | **average** | **100.00** | **96.26** | **82.49** | **62.96** | **49.68** | **23.98** | **10.06** | **0.67** | **-0.54** |
|  | **SEM** |  | **2.47** | **6.23** | **6.10** | **13.24** | **1.57** | **3.10** | **0.23** | **0.50** |
| **A431** |  | 100.00 | 85.11 | 91.34 | 90.38 | 78.84 | 61.28 | 16.64 | 1.24 | 0.90 |
|  |  |  | 92.59 | 97.51 | 95.98 | 88.42 | 62.75 | 21.20 | 1.39 | 1.44 |
|  |  |  | 99.73 | 91.94 | 92.55 | 85.28 | 68.87 | 31.96 | 2.58 | 0.85 |
|  | **average** | **100.00** | **92.48** | **93.59** | **92.97** | **84.18** | **64.30** | **23.27** | **1.73** | **1.06** |
|  | **SEM** |  | **4.22** | **1.96** | **1.63** | **2.82** | **2.32** | **4.54** | **0.42** | **0.19** |
| **LS180** |  | 100.00 | 99.15 | 77.61 | 113.35 | 120.33 | 28.44 | 9.64 | 8.08 | 2.27 |
|  |  |  | 108.15 | 110.99 | 91.46 | 84.08 | 33.47 | 21.52 | 9.56 | 4.64 |
|  |  |  | 80.00 | 93.35 | 58.50 | 54.71 | 48.76 | 26.93 | 10.54 | 3.75 |
|  | **average** | **100.00** | **95.77** | **93.98** | **87.77** | **86.37** | **36.89** | **19.37** | **9.40** | **3.55** |
|  | **SEM** |  | **8.30** | **9.64** | **15.94** | **18.98** | **6.11** | **5.11** | **0.71** | **0.69** |

**Table S3**. In vivo and ex vivo fluorescence imaging data for A549 tumor-bearing mice. (**a**) Mean tumor fluorescence intensity in vivo at 24 h and 48 h after IGN523-IR700 administration, before and after irradiation. (**B**) Organ fluorescence intensity ex vivo after 24 h of IGN523-IR700 administration via i.v. injection.

**a**

|  | **24 h** | | **48 h** | |
| --- | --- | --- | --- | --- |
|  | **Pre irradiation** | **Post irradiation** | **Pre irradiation** | **Post irradiation** |
| **Mouse 1** | 3.37 | 0.98 | 3.38 | 0.72 |
| **Mouse 2** | 4.67 | 0.00 | 2.88 | 0.94 |

**b**

| **Organs** | **Mean Intensity** |
| --- | --- |
| Tumor | 0.97 |
| Kidney | 1.95 |
| blood | 0.09 |
| Spleen | 0.77 |
| Lung | 0.35 |
| Heart | 0.34 |
| Pancreas | 0.32 |
| Stomach | 1.25 |
| Intestine | 0.97 |
| Liver | 3.92 |

**Table S4**. Raw data for Figure 4d. Tumor volume for each A549 tumor-bearing mouse (n ≥ 6) in *in vivo* NIR-PIT study at each time point.

| **Tumor volume (mm^3^)** | | | | | | | | |  |
| --- | --- | --- | --- | --- | --- | --- | --- | --- | --- |
| **Days** |  | **0** | **3** | **6** | **8** | **10** | **13** | **15** | |
| **Control** | mouse 1 | 76.17 | 92.19 | 188.14 | 204.58 | 258.31 | 312.16 | 591.20 | |
|  | mouse 2 | 70.03 | 85.76 | 97.02 | 136.93 | 147.90 | 142.83 | 188.66 | |
|  | mouse 3 | 76.14 | 84.23 | 122.69 | 182.91 | 243.98 | 278.82 | 360.63 | |
|  | mouse 4 | 67.10 | 72.67 | 155.99 | 208.94 | 246.83 | 284.86 | 315.81 | |
|  | mouse 5 | 65.78 | 67.24 | 81.51 | 132.77 | 144.07 | 168.29 | 239.92 | |
|  | mouse 6 | 83.77 | 109.52 | 138.72 | 153.31 | 169.08 | 179.55 | 351.18 | |
|  | mouse 7 | 85.90 | 112.66 | 139.98 | 254.77 | 287.29 | 332.15 | 558.48 | |
|  | **average** | **74.98** | **89.18** | **132.01** | **182.03** | **213.92** | **242.67** | **372.27** | |
|  | **SEM** | **2.97** | **6.48** | **13.54** | **16.77** | **22.14** | **29.04** | **57.24** | |
| **NIR** | mouse 1 | 77.00 | 95.52 | 104.76 | 108.62 | 112.14 | 126.63 | 188.74 | |
|  | mouse 2 | 108.00 | 118.48 | 131.72 | 198.13 | 254.39 | 310.27 | 569.61 | |
|  | mouse 3 | 62.50 | 75.27 | 108.54 | 130.94 | 157.83 | 192.55 | 228.55 | |
|  | mouse 4 | 70.47 | 109.17 | 110.77 | 192.12 | 215.57 | 249.75 | 282.06 | |
|  | mouse 5 | 96.55 | 106.14 | 130.99 | 243.52 | 267.31 | 281.32 | 355.54 | |
|  | mouse 6 | 54.27 | 70.79 | 74.02 | 131.69 | 138.37 | 144.40 | 224.73 | |
|  | mouse 7 | 80.08 | 120.33 | 126.14 | 149.71 | 157.29 | 172.61 | 172.17 | |
|  | **average** | **78.41** | **99.39** | **112.42** | **164.96** | **186.13** | **211.08** | **288.77** | |
|  | **SEM** | **7.09** | **7.49** | **7.65** | **18.05** | **22.63** | **26.58** | **52.26** | |
| **IGN523-IR700** | mouse 1 | 74.86 | 80.35 | 113.49 | 154.87 | 186.56 | 212.35 | 440.53 | |
|  | mouse 2 | 112.93 | 119.74 | 161.79 | 251.54 | 191.68 | 217.43 | 432.76 | |
|  | mouse 3 | 64.25 | 80.11 | 83.12 | 106.87 | 115.05 | 121.18 | 197.15 | |
|  | mouse 4 | 57.75 | 79.71 | 93.70 | 156.25 | 140.72 | 146.38 | 156.93 | |
|  | mouse 5 | 80.67 | 114.21 | 129.81 | 187.66 | 227.49 | 231.15 | 279.99 | |
|  | mouse 6 | 78.39 | 105.35 | 114.96 | 207.76 | 233.11 | 259.84 | 455.92 | |
|  | **average** | **78.14** | **96.58** | **116.15** | **177.49** | **182.44** | **198.05** | **327.21** | |
|  | **SEM** | **12.11** | **14.26** | **18.23** | **28.55** | **27.98** | **30.58** | **60.45** | |
| **PIT** | mouse 1 | 104.90 | 119.45 | 118.52 | 150.34 | 173.71 | 220.06 | 307.51 | |
|  | mouse 2 | 44.02 | 47.13 | 57.80 | 85.16 | 77.38 | 90.32 | 127.25 | |
|  | mouse 3 | 81.72 | 83.69 | 96.70 | 92.35 | 111.25 | 147.19 | 198.52 | |
|  | mouse 4 | 54.22 | 68.00 | 75.71 | 80.19 | 110.86 | 120.30 | 215.18 | |
|  | mouse 5 | 71.94 | 71.94 | 74.99 | 74.14 | 73.79 | 151.62 | 175.26 | |
|  | mouse 6 | 82.52 | 93.38 | 117.45 | 73.60 | 93.41 | 98.37 | 145.26 | |
|  | mouse 7 | 82.19 | 95.88 | 64.65 | 124.31 | 142.92 | 156.55 | 190.59 | |
|  | mouse 8 | 112.55 | 122.32 | 144.73 | 186.94 | 163.68 | 190.22 | 264.49 | |
|  | **average** | **79.26** | **87.72** | **93.82** | **108.38** | **118.37** | **146.83** | **203.01** | |
|  | **SEM** | **8.40** | **9.07** | **11.85** | **15.54** | **12.55** | **13.35** | **17.17** | |
